# Gingiva Mesenchymal Stem Cells Normoxic or Hypoxic Preconditioned Application Under Orthodontic Mechanical Force on Osterix, Osteopontin, and ALP Expression

**DOI:** 10.1055/s-0043-1772699

**Published:** 2023-11-23

**Authors:** Alexander Patera Nugraha, Ida Bagus Narmada, Ervina Restiwulan Winoto, I Gusti Aju Wahju Ardani, Ari Triwardhani, Alida Alida, Adya Pramusita, Reyhan Mahendra Nur, Nuraini Indrastie, Hui Yin Nam, Igo Syaiful Ihsan, Wibi Riawan, Fedik Abdul Rantam, Albertus Putera Nugraha, Tengku Natasha Eleena binti Tengku Ahmad Noor

**Affiliations:** 1 Department of Orthodontics, Faculty of Dental Medicine, Universitas Airlangga, Surabaya, Indonesia; 2 Nanotechnology and Catalysis Research Centre (NANOCAT), Universiti Malaya, Kuala Lumpur, Malaysia; 3 Tissue Engineering Group, Department of Orthopaedic Surgery (NOCERAL), Faculty of Medicine, Universiti Malaya, Kuala Lumpur, Malaysia; 4 Stem Cell Research and Development Center, Universitas Airlangga, Surabaya, Indonesia; 5 Biomolecular Biochemistry, Faculty of Medicine, Universitas Brawijaya, Malang, Indonesia; 6 Laboratory of Immunology and Virology Department of Microbiology, Faculty of Veterinary Medicine, Universitas Airlangga, Surabaya, Indonesia; 7 Faculty of Medicine, Universitas Airlangga, Surabaya, Indonesia; 8 Membership of Faculty of Dental Surgery, Royal Collage of Surgeon, Edinburgh University, United Kingdom; 9 Malaysian Armed Forces Dental Officer, 609 Armed Forces Dental Clinic, Kem Semenggo, Kuching, Sarawak, Malaysia

**Keywords:** alveolar bone, hypoxia, medicine, orthodontic tooth movement, stem cells

## Abstract

**Objectives** The aim of this article was to investigate Osterix, ALP, and osteopontin expression in the compression and tension sides of alveolar bone after the application of normoxic/hypoxic-preconditioned GMSCs in rabbits ( *Oryctolagus cuniculus* ) induced with OMF.

**Materials and Methods** Forty-eight healthy, young male rabbits were divided into four groups: [-] OMF; [+] OMF; OMF with GMSCs normoxic-preconditioned; and OMF and GMSCs hypoxic-preconditioned. The central incisor and left mandibular molar in the experimental animals were moved, the mandibular first molar was moved mesially using nickel titanium (NiTi) and stainless steel ligature wire connected to a 50 g/mm ^2^ light force closed coil spring. Allogeneic application of normoxic or hypoxic-preconditioned GMSCs was used in as many as 10 ^6^ cells in a 20 µL phosphate buffered saline single dose and injected into experimental animals' gingiva after 1 day of OTM. On days 7, 14, and 28, all experimental animals were euthanized. Osterix, ALP, and osteopontin expressions were examined by immunohistochemistry.

**Results** Osterix, ALP, and osteopontin expressions were significantly different after allogeneic application of hypoxic-preconditioned GMSCs than normoxic-preconditioned GMSCs in the tension and compression of the alveolar bone side during OMF ( *p* < 0.05).

**Conclusion** Osterix, ALP, and osteopontin expressions were significantly more enhanced post-transplantation of GMSCs with hypoxic-preconditioning than after transplantation of normoxic-preconditioned GMSCs in rabbits ( *O. cuniculus* ) induced with OMF.

## Introduction

Orthodontic tooth movement (OTM) is defined as a biological response to the disruption of the dentofacial complex's physiological balance due to an applied external mechanical force. 1 The direction of force where bone remodeling occurs—specifically, bone resorption on the compression side and bone apposition on the tension side of the periodontal ligament (PDL)—determines the direction in which teeth move. 2 The ideal strength for orthodontic mechanical force (OMF) is the amount of force that can promote cellular activity without harming the blood vessels in the PDL. 3 Tooth movement occurs in three stages: (1) changes in blood flow caused by pressure on the PDL, (2) the creation and/or release of chemical mediators, and (3) cell activation. 4 5

The expanding number of adult orthodontic patients has resulted in increased demand for shorter treatment periods, enhanced periodontal health, and long-term stability. Various techniques for quickening nonsurgical and surgical orthodontic therapy have been tried and tested for success. Frost presented the idea of a regional acceleration phenomena (RAP) in 1989, which explains the physiologic basis of fast tooth movement after bone fracture. RAP is a local reaction to damage, which varies in length, size, intensity, and number of stimuli and can last for 4 to 6 months in human bone. Bone turnover in some areas is 10 to 50 times quicker than in normal bone. The orthodontic accelerated periodontal therapy (PAOO) concept was launched in 2001. Orthodontic tooth movement (OTM) can be encouraged through surgical techniques such as corticotomy, micro-osteoperforation, decortication, and osteotomy to enhance PAOO, but patients may decline these due to concerns about the morbidity associated with orthodontic surgery. Thus, patients may choose nonsurgical methods to accelerate OTM. The time for comprehensive orthodontic treatment varies greatly, but current best evidence based on prospective studies done in a university research context shows that full treatment takes less than 2 years on average to complete. 6 Several factors, such as case severity, extraction or nonextraction approach, clinical expertise, and patient cooperation, can influence the length of treatment. For example, studies have shown that correction of Class II associations takes approximately 5 months longer than Class I occlusions with distance severity. 7

It is still doubtful that stable and optimal treatment results will be obtained despite implementation of nonsurgical interventions for OTM acceleration, such as the modification of different variations in self-ligating bracket designs, chemical and herbal compound permeation, microvibration, low-intensity lasers, photobiomodulation, and pulsed electromagnetic fields. 8 Therefore, the use of mesenchymal stem cells (MSCs) in orthodontic therapy may be promising. Stem cells are self-renewing cells that, in the correct circumstances, may differentiate themselves into a wide range of cells. 9 Muscle, dermis, bone marrow, adipose tissue, periosteum, blood, umbilical cord, synovial membranes, and teeth are just a few of the tissues from which MSCs can be consumed. 10 11 The capacity of MSCs generated from dental pulp, PDL, or exfoliated human primary teeth to differentiate and multiply has been demonstrated in several studies. 12 13 14 15

MSCs are supplied through an osteoconductive scaffold and transformed into osteogenic cells that employ osteoinductive growth factors in bone tissue engineering. 16 There have been several scaffolds and growth factors used to repair craniofacial bone abnormalities, including bone deformities caused by orthodontic therapy. 17 18 19 It is crucial to present the potential advantages of stem cell therapy for increasing orthodontic treatment and the use of stem cells to treat dentofacial anomalies and deformities. As a result, current regenerative dentistry research about using stem cells to extend the limitations of regular OTM, OTM with periodontal abnormalities, expedited OTM, and external root resorption therapy must be explored when assessing the use of stem cells in orthodontics. 20

Mechanical load-driven remodeling of the PDL and alveolar bone is caused by OMF. 1 2 3 The PDL microvessels' constriction causes localized necrosis, which sets off the initial inflammatory response at the compression site. Next, osteoclasts are recruited from the surrounding bone marrow region. 21 The vast majority of these osteoclasts is produced by hematopoietic stem cells. 22 Stem cells have the ability to accelerate OTM in response to orthodontic pressures. When orthodontic pressures are utilized, tooth movement is delayed until the necrosis is eliminated, resulting in a clinical lag period. Theoretically, stem cell transplantation brought on by stress may hasten the procedure, leading to quicker OTM. 20

Gingiva is a distinct tissue that is part of the periodontal tissue surrounding the teeth and alveolar bone. When the gingiva is injured, it has the potential for adequate repair without scarring. The capacity of gingival wounds to regenerate and mend is substantially faster than typical healing, lasting 7 to 14 days compared to the 14 to 21 days required for skin tissue. This demonstrates that the gingival tissue contains a sufficient stem cell pool and source for tissue regeneration. 23 24 However, the possibility of contamination when isolating GMSCs should be considered. Before isolating GMSCs from the patient's gingiva, tartar must be removed, and antiseptic mouthwash must be administered as a decontamination treatment. To decrease the danger of contamination, a GMSC culture must be completed in a sterile environment and according to procedures. 23

MSC treatment relies heavily on transplants. The clinical trial study has indicated little engraftment capacity following transplantation of MSCs that are cultivated in ambient settings. It has previously been observed that bone marrow mesenchymal stem cells (BM-MSCs) fail to engraft into nonhematopoietic tissue *in vivo* , but this problem may be solved using a variety of methods. Furthermore, MSCs that are conditioned in a hypoxic environment have shown increased skeletal muscle regeneration at day 7 as well as increased blood flow and vascular formation when compared to MSCs maintained in normoxic conditions. 25 Expression of the chemokine receptor motif C-X-C Receptor-4 (CXCR-4) and CXC Receptor-7 motif (CXCR-7) is upregulated when MSCs are exposed to hypoxia. 26 These chemokine receptors are critical for MSC migration in damaged tissues. 25

The existence of a danger-associated molecular pattern (DAMP) was linked to the onset of sterile inflammation as well as the activation of mechanical force damage after the application of fixed orthodontic equipment during OTM. DAMPs are endogenous chemicals that are selectively and rapidly produced under cellular stress, damage, or necrosis, which are circumstances that cause host tissue inflammation. Many endogenous chemicals such as DNA, ATP, uric acid, DNA-binding proteins, and reactive oxygen species are produced after trauma or damage that is caused by the inflammatory response. Infected or repressed host cells emit DAMPs. These endogenous chemicals connect to particular DAMP receptors (DAMPRs), resulting in the release of cytokines and chemokines such as interleukin (IL)-1, IL-6, and tumor necrosis factor-α which have a significant role in osteoimmunology. Endothelial dysfunction can result from cytokines, neutrophil, and monocyte transmigration, and vasodilation and increased capillary permeability. During cellular stress, resolution-associated molecular patterns (RAMPs) are released. RAMPs work to counteract the inflammatory effects of pathogen-associated molecular pattern (PAMP) and DAMP. RAMPs decrease macrophage activity, resulting in the resolution of inflammation either directly by antagonizing PAMP (e.g., heat shock protein (HSP)-10) or indirectly by inducing IL-10 and other anti-inflammatory cytokines, which result in cell differentiation. 27

Based on the foregoing, we sought to investigate whether there were any variations in the expression of Osterix, osteopontin, ALP, and alveolar bone pressure following allogeneic transplantation of hypoxic or normoxic preconditioned GMSCs into male rabbits ( *Oryctolagus cuniculus* ) in an *in vivo* experiment.

## Materials and Methods

### Ethical Permit for Animal Experiment

This study obtained an ethical permit for conducting an animal experiment in the dental medicine field with appointment number 683/HRECC.FODM/IX/2022,83/HRECC.FODM/IX/2022, which established the proper standards and guidelines that were followed throughout this investigation.

### Experimental Study Design

Forty-eight healthy, male, six-month-old *Oryctolagus cuniculus* rabbits with 3 to 4 kg body weights and complete teeth and healthy periodontium were used in this experiment as animal models. Each rabbit spent a week in a cage measuring 60 × 40 × 40 cm before treatment. Each animal was housed in a temperature-controlled environment (22–24°C) with a 12-hour cycle of darkness and light, and each rabbit had access to food and drink in accordance with the standards. This research has four experimental groups, namely [-]OMF; [+]OMF, OMF with GMSCs normoxic-preconditioned, and OMF and GMSCs hypoxic-preconditioned. The application of the treatment was performed in the affected mandibular gingiva using a sharp microneedle (Hamilton microneedle syringe, Sigma-Aldrich, St. Louis, MO, United States) and a local infiltration approach in the gingival sulcus. Allogeneic application of GMSCs normoxic or hypoxic-preconditioned 10 ^6^ cells in 20 µL phosphate buffered saline (PBS) single dose was injected into experimental animals' gingiva after 1 day of OMF.

### Orthodontic Mechanical Force Installation

Regarding the central incisor and left mandibular molar in the experimental animals, we moved the mandibular first molar mesially by means of a 0.07 stainless steel ligature wire (MicoOne, Taipei, Taiwan) that fastened the fixed orthodontic device: a 50 g/mm ^2^ light force closed coil spring (MicoOne, Taipei, Taiwan). The calibration of each OMF animal model was examined with a tension gauge (MicoOne, Taipei, Taiwan) to maintain the OMF light force. An intravenous anesthetic (3% pentobarbital sodium, 1 mL/kg) was given to the animals to lessen their discomfort over the course of treatment. 28 Histomorphometric observation was done directly in the OMF animal model to establish whether OMF induced tooth movement.

### Culture, Subculture, Confirmation, Hypoxia Preconditioning, and Implantation of Gingiva Mesenchymal Stem Cells

The GMSC isolation procedure was completed in accordance with the results of the prior study. 29 The free margin of the gingiva of the rabbits was removed with a sharp blade and then minced into bits. The cell culture media was supplemented with Dulbecco's Modified Eagle Medium (Thermo Fisher Scientific, Inc., Waltham, MA, USA) with a 20% fetal bovine serum (Sigma-Aldrich, St. Louis, MO, United States), 5 mL glutamine (StemCells Corporation, United States), 100 U/mL penicillin-G, 100 g/mL streptomycin, and 100 g/mL kanamycin (Sigma-Aldrich, St. Louis, MO, United States).

Cultured GMSCs with 80% cell confluence were passaged once with trypsin-ethylenediaminetetraacetic acid (EDTA) and then washed and grown in tissue culture plates. The fourth passage of GMSCs were induced by cobalt chromium II (CoCl _2_ ) as a hypoxia mimicking agent with a concentration of up to 100µmol to achieve cellular hypoxia precondition. 30

### Immunohistochemistry Examination

The sample of each rabbit's mandible was cut and placed in the 10% neutral buffer formalin (Sigma-Aldrich, St. Louis, MO, United States) tissue fixation solution. In addition, each rabbit's mandible underwent the decalcification process by mean of EDTA (Sigma-Aldrich, St. Louis, MO, United States) for 4 months. The rabbits' mandibula were washed with running tap water for approximately 10 to 15 minutes. After cleaning, the mandibula was dried before being washed 3 times for approximately 5 to 10 minutes each time with 1x PBS (OneMed, Indonesia), which was followed by drying. The sample was then fixed for 4 to 7 days in a 10% neutral buffer formalin (Sigma-Aldrich, St. Louis, MO, United States). The mandibula was then cleansed three times for 5 to 10 minutes each with 1x PBS (OneMed, Indonesia). To decalcify the calvaria, EDTA was used for 14 to 28 days. After softening the mandibula, the histology pathology anatomy (HPA) slide was made through tissue processing, embedding, and sectioning using a microtome.

To count the number of Osterix, osteopontin, and ALP-positive expressions in the osteoblast in the animal models' alveolar bone, a 1:500 dilution of Osterix (OSX antibody [F-3]: sc-393325), osteopontin (osteopontin/OPN/SPP1 antibody [AKm2A1]: sc-21742), ALP (A-10: sc-271431), and monoclonal antibody was utilized (Santa Cruz Biotechnology Inc., Texas, United States). Following that, diaminobenzidine was applied, producing a brown precipitate that formed where the antibody had adhered, which was counterstained on the HPA slide with hematoxylin eosin. The number of positive Osterix, osteopontin, and ALP expressions in osteoblasts in the alveolar bone on the tension and compression side was determined using an inverted light microscope (Nikon, Tokyo, Japan) at 400x and 1000x magnification in five fields of view that were examined by two observers. The data acquired was then recapitulated and analyzed.

### Statistical Analysis

Following the summary, descriptive and inferential analysis of the research data was conducted. The mean and standard deviation of the data are shown in a bar chart. The data was analyzed using one-way analysis of variance along with a Tukey's honest significance different test to compare the difference between groups ( *p* < 0.05). The Statistical Package for Social Science version 23.0 for Windows was used in this study (IBM corporation, Illinois, Chicago, United States).

## Results

**Fig. 1:**
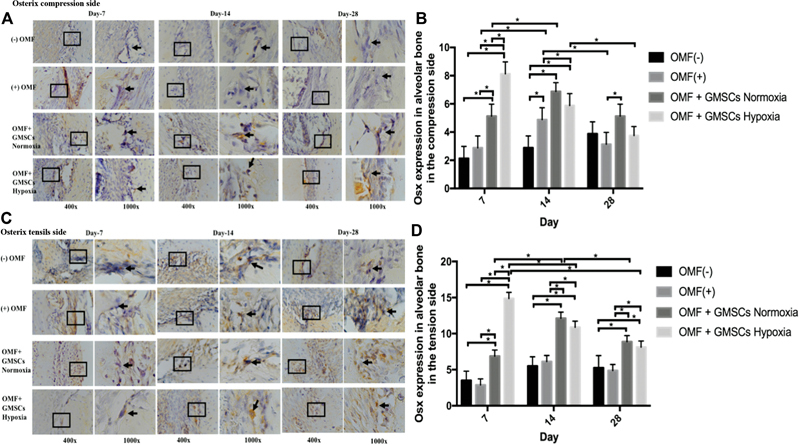
Using a light-inverted microscope at magnifications of 400x and 1000x, the positive expression of Osterix in the osteoblast in the alveolar bone of tension ( **A** ) and compression ( **C** ) side in each group was seen. Day 7, day 14, and day 28 showed positive osteoblast expression in both the compression ( **B** ) and tension ( **D** ) sides in each group, which was significant across groups ( *p* < 0.05). *Information: significant difference between groups at *p* -value less than 0.05. GMSCs, gingiva mesenchymal stem cells; OMF, orthodontic mechanical force.

**Fig. 2:**
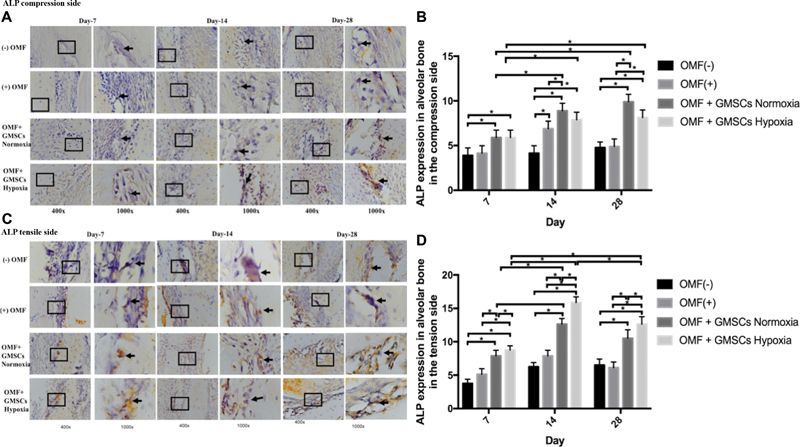
Using a light-inverted microscope at magnifications of 400x and 1000x, the positive expression of alkaline phosphatase (ALP) in the osteoblast in the alveolar bone of the tension ( **A** ) and compression ( **C** ) sides in each group was seen. Day 7, day 14, and day 28 showed positive ALP expression in both the compression ( **B** ) and tension ( **D** ) sides in each group, which was significant across groups ( *p* < 0.05). *Information: significant difference between groups at *p* -value less than 0.05. GMSCs, gingiva mesenchymal stem cells; OMF, orthodontic mechanical force.

**Fig. 3:**
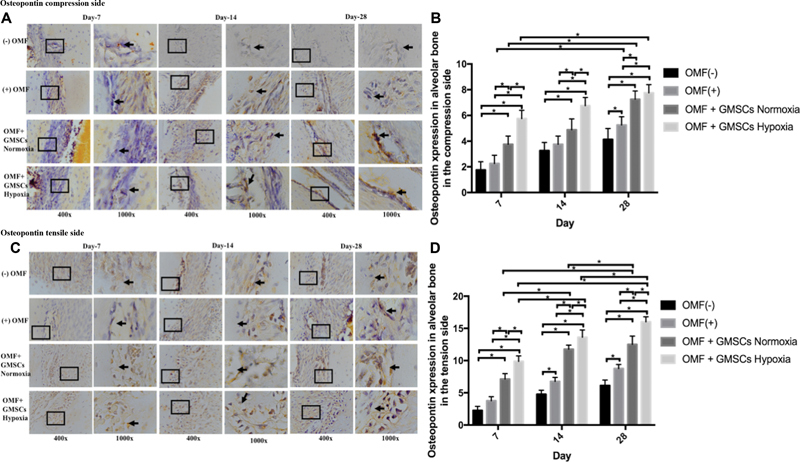
Using a light-inverted microscope at magnifications of 400x and 1000x, the positive expression of osteopontin in the osteoblast in the alveolar bone of the tension ( **A** ) and compression ( **C** ) sides in each group was seen. Day 7, day 14, and day 28 showed positive osteopontin expression in both the compression ( **B** ) and tension ( **D** ) sides in each group, which was significant across groups ( *p* < 0.05). *Information: significant difference between groups at *p* -value less than 0.05. GMSCs, gingiva mesenchymal stem cells; OMF, orthodontic mechanical force.

Upon histological analysis, positive expression of Osterix, ALP, and osteopontin was detected in all groups ([-] OMF; [+] OMF, OMF + GMSCs Normoxia, and OMF + GMSCs hypoxia) on day 7, 14, and 28. Positive expression of Osterix, osteopontin, and ALP was identified in alveolar bone osteoblasts on the tension and compression side ( Figs. 1A, C ; 2A, C ; 3A, C ). The peak expression of Osterix was found on day 7. In addition, Osterix expression was higher in OMF + GMSCs hypoxia on day 7 compared to OMF + GMSCs normoxia or [+] OMF and [-] OMF. Osterix expression was significantly different between the tension and compression sides of alveolar bone ( *p* < 0.05; Fig. 1B and 1D ). The peak expression of ALP was found on day 14. In addition, ALP expression was higher in OMF + GMSCs hypoxia on day 14 compared to OMF + GMSCs normoxia or [+] OMF and [-] OMF. ALP expression was significantly different between the tension and compression sides of alveolar bone ( *p* < 0.05; Fig. 2C and D ). The peak expression of osteopontin was found on day 14. In addition, Osterix expression was higher in OMF + GMSCs hypoxia on day 14 compared to OMF + GMSCs normoxia or [+] OMF and [-] OMF. Osteopontin expression was significantly different between the tension and compression sides of alveolar bone ( *p* < 0.05; Fig. 3C and D ).

## Discussion

The application of orthodontic forces for malocclusion treatment will begin cellular and molecular signaling and regulation in the periodontal tissue. Fibroblasts, osteoblasts, osteoclasts, and macrophages are types of cells that play an interactive role in bone remodeling.

On the compression side, bone resorption occurs due to osteoclast activity that originates from hematopoietic stem cells on the compression side, and bone apposition occurs due to osteoblast activity that originates from MSCs on the tension side during OMF. 2 The goal of this study was to investigate whether transplanting allogeneic GMSCs preconditions hypoxia to Osterix, ALP, and osteopontin expression on the tension and compression sides. Those bone apposition biomarkers are important for the investigation of alveolar bone remodeling under OMF. 1 3 Optimal OTM rate can be achieved if there is homeostasis between bone apposition and bone resorption. 3 4

On day 7, the maximum expression of Osterix was reported in the OMF + GMSCs hypoxia. Osterix is a transcription factor involved in osteoblast development from MSCs and osteogenesis. 31 *In vivo* , fluid tension, compressive, and shearing forces activate Osterix, and mechanical stresses such as compressive, tension, and shearing forces have been shown to increase Osterix expression in osteoblast lineage cells and promote osteoblast differentiation *in vitro* . Osterix has the ability to modulate mechanotransduction in osteoblast cells in order to promote bone growth. 31 32 33 Osterix's effect on cell proliferation may vary depending on the stage of osteoblast development. Several investigations have shown that mechanical stress activates Osterix in osteoblast cells, promoting osteogenesis. 34 35 This finding is consistent with the previous study, which found that orthodontic force regulated the increased expression of RUNX2 and Osterix at the mRNA and protein levels during the 7-day experimental period when compared to the control group, and the upregulation increased with increasing experimental time, peaking at day 14. In mice that were not given OMF, however, there was no change in RUNX2 or Osterix expression. These findings show that RUNX2 and Osterix may be involved in bone cells' early responsiveness to mechanical cues. 36

In periodontal tissues, GMSCs are multipotent stem cells that can develop into osteoblast-like cells by producing proteins that express the phenotype and function of osteoblasts under external mechanical pressures. Osteoblast-specific transcription factors are important in this process. Biologically, mechanical stimulation can stimulate and begin the differentiation of MSCs into bone cells and control bone cell maturation. Osteocalcin expression can be regulated by Osterix to enhance osteoblast maturation. Osterix is also thought to be important in the control of bone cell development. 29 37

On day 28, the OMF + GMSCs hypoxia had the greatest osteopontin expression. The expression of osteopontin differed significantly across groups. One of the most prevalent noncollagenous proteins in the periodontium is osteopontin. The protein accounts for up to 3% of total bone protein and includes 49 amino acids, three of which are gamma-carboxyglutamic acid residues with calcium-binding capabilities. Osteocalcin is generated by PDL osteoblasts, cementoblasts, and fibroblasts and is released in tissues for mineralization. 38 In animal models treated with warfarin, a vitamin K antagonist, osteopontin synthesis was studied, and carboxylation of glutamic acid residues was inhibited. Osteopontin regulates the development of hydroxyapatite crystals and the recruitment of osteoclasts that engage in bone remodeling throughout the biomineralization process as well as in the extracellular matrix. 39

The expression of the osteopontin gene during OMF may be linked to the activation of early bone and matrix resorption of PDL collagen fibers, which is essential for OMF and the particular recruitment of osteoclasts at the resorption site. During OMF, the expression of the gene producing osteocalcin remains elevated. 40 It has been proven that the microenvironment present during the application of orthodontic pressures during OMF, as well as local tissue inflammation, influences the outcome of wound healing and regeneration of periodontal tissues. 41 42

MSCs are often cultivated *in vitro* in normoxia (21% O _2_ ), yet they are frequently found in a low oxygen environment *in vivo* : 1 to 7% is found in bone marrow, 10 to 15% in adipose tissue, and 2.5 to 8.5% in the placenta. 43 *In vivo* , MSCs play a crucial role in bone healing in a low oxygen environment. 44 Oxygen stress is a well-known signaling molecule that affects the proliferation and differentiation of MSCs. It has been reported that hypoxia can increase osteogenic differentiation of human placental MSCs. After cultivating hypoxia-preconditioned MSCs, the expression of osteogenic genes such as osteopontin, osteocalcin, and ALP increased considerably. Under hypoxic preconditions, total calcium deposition and ALP levels increased significantly, confirming that hypoxic preconditions induce osteogenic differentiation of human placental MSCs. 45 Previous research has demonstrated that 2% O _2_ stress regulates the expression of osteocalcin and osteopontin. 46 Furthermore, hypoxia can enhance MSC and ALP activity. 47 Previous findings suggest that hypoxia preconditioning increases osteogenic differentiation of MSCs. Hypoxic preconditioning promotes osteogenic differentiation of MSCs in the early phases, but it inhibits osteogenesis in the later stages. 48

CoCl _2_ has been frequently employed in hypoxic preconditioning studies, both *in vitro* and *in vivo* . 49 CoCl _2_ was also employed in this investigation to precondition hypoxia for allogeneic GMSCs to be transplanted during OMF. Previous research employed CoCl _2_ to precondition hypoxia in human periodontal ligament cell (hPDLC) by stabilizing HIF-α. CoCl _2_ -induced hypoxia preconditioning increased osteogenic differentiation features such as ALP expression, mineralization activity, mRNA expression, and protein levels of osteogenic genes such as Osterix, ALP, Osteocalcin, and osteopontin in hPDLC. 50 51 The degree of severity and duration of hypoxia, the isolation protocol, and the mechanism utilized to induce hypoxic preconditioning in MSCs all influence hypoxic preconditioning in MSCs. 30

The area of tissue injury on the tension side differs from that on the compression side during OMF. On the tension side, the majority of osteopontin is found in the cytoplasm of osteoblasts, cementoblasts, and PDL cells, whereas on the pressure side, it is found in the extracellular matrix of the periodontal membrane. The findings of this investigation support prior findings by demonstrating that OMF stimulation of periodontal tissues might promote osteoblast growth or differentiation. 52

Osteopontin is considered a marker of osteoblast differentiation because it is expressed in the cells of the osteoblast lineage both *in viv* o and *in vitro* . Osteopontin is a biomolecular marker of mature osteoblast activity during bone apposition that is produced by osteoblasts that may bind to hydroxyapatite and is related to bone matrix mineralization. In the perinuclear arrangement that lines up in active bone surface creation, cuboid cells display a very high positive signal for osteocalcin. Under OMF, osteogenic cells in the PDL express osteocalcin. Based on changes in osteopontin expression, it was proposed that osteopontin plays a role in active bone remodeling, with substantial bone resorption on the stress side and bone deposition on the tension side under OMF. The peak of osteopontin expression was found to be a predictor of bone formation time in this investigation. Aside from osteopontin, numerous additional bone matrix proteins, such as osteonectin, play key roles in bone remodeling. 51 In individuals with primary hip osteoarthritis, osteoblast markers such as osteocalcin, osteopontin, and Col1a1 mRNA were shown to be enhanced. 53 Increased expression of ALP and Col1a1 mRNA characterizes the early stage of osteoblastogenesis through preosteoblasts. Early osteoblasts express osteopontin, which is distinguished by its capacity to bind to hydroxyapatite. 54 Previous research revealed that alveolar bone under OMF resulted in a substantial rise in ALP and Col1a1 mRNA gene expression from day 2 to 6. 55

The application of MSCs to PDL cells or direct MSC activity in PDL tissues may have an accelerated impact on OTM by expediting the bone remodeling process via osteoblast-osteoclast turnover rate. Furthermore, MSC cells may lower collagen I expression with OTM and regain collagen synthesis after force removal, a process that was previously interpreted as enhancing MSCs' putative OTM accelerator capacity. 56 The metabolic demand may encourage MSCs to quicken bone remodeling, allowing tooth movement with minimum or no necrotic hyalinized tissue, which causes tooth movement delay under OMF. 56

GMSCs hypoxic preconditioning was applied to both sides. Compression and tension were hypothesized to accelerate bone remodeling during OMF based on the concept of delay period prevention that is induced by hyalinized necrotic tissue removal and increased wound healing rate. The use of GMSCs most likely increased the replenishment, activation, and/or differentiation of periodontal MSCs, which developed into reparative cells such as osteoblasts or cementoblasts. However, this study limitations are that this study involved only an immunohistochemical investigation, which was one shortcoming of this research. The expression of bone molecular markers may be assessed using methods like quantitative reverse transcription-polymerase chain reaction and/or western blot analysis to investigate mRNA level related bone remodeling and molecular pathway of bone remodeling in future studies. An effort should be made to determine the changes in alveolar bone and periodontal tissue after hypoxia-preconditioned GMSC transplantation into OMF animal models with a longer observation period.

## Conclusion

Enhancement Osterix, ALP, and osteopontin expression in the tension and compression side of alveolar bone under OMF was found after transplantation of hypoxic-preconditioned GMSCs was significantly different than GMSCs with normoxic preconditioning. Future study is needed to compare the various GMSC preconditioning methods, such as comparing normoxia, hypoxia, or hyperoxia with various examination methods within an *in vitro* or *in vivo* setting before it can be applied in clinical setting study.
